# Risk of Hospital Readmissions and Association With Receipt of Post‐Hospitalization Care Coordination Services Among High‐Risk Veterans

**DOI:** 10.1111/1475-6773.70044

**Published:** 2025-09-26

**Authors:** Diana J. Govier, Meike Niederhausen, Alex Hickok, Mazhgan Rowneki, Holly McCready, Abby Moss, Kristina M. Cordasco, Kathryn M. McDonald, Matthew L. Maciejewski, Kathleen C. Thomas, Denise M. Hynes

**Affiliations:** ^1^ VA HSR Center to Improve Veteran Involvement in Care (CIVIC) VA Portland Health Care System Portland Oregon USA; ^2^ Veterans Rural Health Resource Center—Portland, Office of Rural Health, Veterans Health Administration Portland Oregon USA; ^3^ Oregon Health & Science University—Portland State University School of Public Health, Oregon Health & Science University Portland Oregon USA; ^4^ VA Office of Geriatrics and Extended Care, Veterans Health Administration Washington DC USA; ^5^ VA HSR Center for the Study of Healthcare Innovation, Implementation & Policy (CSHIIP) VA Greater Los Angeles Healthcare System Los Angeles California USA; ^6^ Department of Medicine David Geffen School of Medicine at the University of California Los Angeles California USA; ^7^ Center for Diagnostic Excellence, Armstrong Institute for Patient Safety and Quality, Johns Hopkins School of Nursing Baltimore Maryland USA; ^8^ Division of General Internal Medicine, Department of Medicine Johns Hopkins School of Medicine Baltimore Maryland USA; ^9^ VA HSR Center of Innovation to Accelerate Discovery and Practice Transformation (ADAPT), Durham Veterans Affairs Health Care System Durham North Carolina USA; ^10^ Department of Population Health Sciences & Division of General Internal Medicine, Department of Medicine Duke University Durham North Carolina USA; ^11^ Division of Pharmaceutical Outcomes and Policy University of North Carolina at Chapel Hill Chapel Hill North Carolina USA; ^12^ School of Nursing, Oregon Health & Science University Portland Oregon USA; ^13^ College of Health & Center for Quantitative Life Sciences, Oregon State University Corvallis Oregon USA

**Keywords:** ambulatory care sensitive condition, care coordination, care management, competing risk, hospital readmission, veterans

## Abstract

**Objective:**

To examine associations between receipt of post‐hospitalization care coordination and VA‐delivered, VA‐purchased, and Medicare fee‐for‐service hospital readmissions among Veterans at high risk for hospitalization and/or mortality.

**Study Setting and Design:**

In this observational retrospective cohort study, we compared high‐risk Veterans who received care coordination within one day after hospital discharge (“treated”) with up to five matched high‐risk Veterans who did not receive care coordination during this time (“comparators”). Competing risk models estimated adjusted sub‐hazard ratios (aSHR) for 30‐day all‐cause and ambulatory care sensitive condition (ACSC) readmissions between treated and comparators, with death as a competing risk. In sensitivity analyses, we implemented inverse probability of censoring weights to account for censoring due to cross‐over to treatment among comparators during follow‐up.

**Data Sources and Analytic Sample:**

Data sources included the VA Vital Status File, VA Corporate Data Warehouse, and Centers for Medicare and Medicaid Services administrative files. Participants included 31,614 treated and 99,634 comparator high‐risk Veterans initially hospitalized in fiscal year 2021.

**Principal Findings:**

Participants were primarily male sex, ≥ 65 years of age, and had initial hospitalizations in VA facilities; 15.9% and 2.3% of treated Veterans had 30‐day all‐cause and ACSC readmissions, respectively, compared with 13.5% and 2.1% of comparators. After accounting for the competing risk of death and covariates that remained imbalanced across groups after matching, post‐hospitalization care coordination was associated with no difference in the risk of 30‐day all‐cause (aSHR 1.03, 95% CI 1.00, 1.07) and ACSC (aSHR 0.97, 95% CI 0.89, 1.05) readmission among high‐risk Veterans. The risk of ACSC readmission was similar after including censoring weights (aSHR 1.00, 95% CI 0.92, 1.09); the increased risk of all‐cause readmission was small in magnitude but statistically significant (aSHR 1.09, 95% CI 1.05, 1.13).

**Conclusions:**

Receipt of post‐hospitalization care coordination was largely associated with no difference in 30‐day readmission risk, suggesting that alternative or additional services may be needed to address readmissions among high‐risk Veterans.

**Summary**

- What is known on this topic?
  ○ Veterans enrolled in the Veterans Health Administration (VA) frequently experience hospital readmissions, which are costly and can lead to poor care experiences and health outcomes.
  ○ Evidence from other patient populations suggests post‐hospitalization care coordination can reduce hospital readmissions; yet, VA has no standard post‐hospitalization care coordination practice for Veterans transitioning home from the hospital.
  ○ Existing VA interventions, which tend to emphasize enhanced primary care models and services, have had little to no effect on hospital readmissions and may or may not include care coordination.
- What this study found?
  ○ Among Veterans at high risk for hospitalization or mortality (“high‐risk Veterans”), receipt of post‐hospitalization care coordination was associated with no difference in the risk of 30‐day all‐cause hospital readmission.
  ○ There was also no difference in the risk of 30‐day ambulatory care sensitive‐condition (ACSC; i.e., potentially avoidable) hospital readmission between high‐risk Veterans who did and did not receive post‐hospitalization care coordination.
  ○ Efforts to enhance care coordination data and measurement are needed to more fully understand its effects on hospital readmissions, as are approaches for reducing avoidable readmissions among high‐risk Veterans.

## 1 Introduction

Hospital readmissions account for a disproportionate share of hospital stays and costs in the US [1]. Strategies to improve timely access to high‐quality post‐hospitalization care have been a focus of US health care reform. In 2013, the Centers for Medicare and Medicaid Services expanded payment for transitional care management services if they occur within seven (for highly complex patients) or 14 (for moderately complex patients) days after discharge [2]. From 2013 to 2018, Medicare spent nearly $1 billion on such post‐hospitalization services [3], a payment change that was associated with increased timely follow‐up after discharge among Medicare fee‐for‐service (FFS) beneficiaries [4]. Receipt of transitional care management services within 30 days of discharge has also been associated with lower total health care costs and mortality at 60 days among Medicare FFS beneficiaries [5].

The Veterans Health Administration (VA) is the largest national integrated health system in the US and serves Veterans with a variety of medical and psychiatric illnesses that place them at risk for hospitalization and readmission [6, 7]. Indeed, approximately 15%–20% of Veterans who are initially hospitalized with high‐risk medical conditions and nearly 25% who are initially hospitalized with chronic psychiatric conditions experience readmission within 30 days [8]. The cost of such readmissions to the VA was $1.2 billion in 2011 [8]. As such, VA has undertaken efforts to integrate care coordination and management into service delivery, with a focus on Veterans with clinical and social factors that place them at risk for hospitalization [9]. Despite this, there is no standard care coordination practice for high‐risk Veterans transitioning home from the hospital [10] and existing VA interventions, which largely emphasize primary care, have had little to no effect on hospital readmissions [11, 12, 13, 14].

Primary care services may or may not include care coordination. Comprehensive care coordination includes patient risk and needs assessment, care planning and education, and communication between patients, care partners, and providers; ideally, care coordination also facilitates integration across the continuum of care and linkages between health system and community resources [15, 16]. Care coordination, broadly, has proven effective for improving clinical outcomes [17, 18, 19] and experiences of care [18, 20, 21, 22], and decreasing or maintaining health care costs [22, 23], including among Veterans at VA facilities [20, 22, 24]. Yet, the value of delivering care coordination services to high‐risk Veterans during or soon after hospital discharge remains largely unknown. Understanding the association between post‐hospitalization care coordination and hospital readmission may help VA to design more effective hospital transition practices for high‐risk Veterans. In this study, we compared the risk of 30‐day all‐cause and ambulatory care‐sensitive condition (ACSC) hospital readmissions in VA‐delivered, VA‐purchased, and Medicare FFS settings among matched cohorts of high‐risk Veterans who did and did not receive VA care coordination services within one day after hospital discharge.

## 2 Methods

### 2.1 Overview

This was an observational retrospective cohort study. Veterans who received care coordination within one day after hospital discharge (“treated”) were matched with up to 5 Veterans who did not receive care coordination during this period (“comparators”) across a variety of variables that may be associated with receipt of care coordination and hospital readmission. Among treated Veterans, only the first instance of post‐hospitalization care coordination was considered. Matched treated Veterans and comparators were then followed for 30 days to compare their risks of all‐cause and ACSC readmission. The VA Portland Health Care System Institutional Review Board approved this study.

### 2.2 Role of the Funding Source

The U.S. Department of Veterans Affairs had no role in the design or conduct of the study; collection, management, analysis, or interpretation of the data; preparation, review, or approval of the manuscript; or the decision to submit the manuscript for publication.

### 2.3 Data and Participants

Data for this study came from the VA Corporate Data Warehouse, which contains information on hospitalizations in VA‐delivered and VA‐purchased care settings, as well as data on Veterans' demographic, geographic, and clinical characteristics, and their health care use. Data on Medicare FFS hospitalizations, and Medicare FFS and Medicare Advantage enrollment, came from the VA‐linked Centers for Medicare and Medicaid Services (CMS) enrollment files and Medicare FFS claims files [25, 26]. Information on Veterans' dates of death came from the VA Vital Status File, VA Corporate Data Warehouse, and CMS files.

The study population included Veterans who were enrolled in VA care during fiscal year (FY) 2021 (October 2020–September 2021) who were categorized as being at high risk for hospitalization and/or mortality and who were discharged alive from a hospital admission during FY2021 (*N* = 379,091). Veterans' high‐risk status was ascertained using the Care Assessment of Need (CAN) score. The CAN score is a VA‐derived measure of a Veteran's percentile risk for hospitalization or death within the next year, based on a variety of clinical diagnoses, health care use metrics, medications, laboratory test values, and vital sign measurements [27]. Veterans with a score of 85 or higher during the year prior to their initial hospitalization were considered high risk. Veterans were excluded from the study for: residence outside the continental US, residing more than 180 days in an inpatient setting or skilled nursing facility during the year prior to initial hospitalization, residing 30 days or more in a skilled nursing facility during the 6 months prior to initial hospitalization, discharge from initial hospitalization to a skilled nursing facility, death prior to discharge from initial hospitalization, and missing covariates used in matching (see ‘Matching’). A total of 52,103 Veterans were excluded, resulting in a pre‐match cohort of 326,988 high‐risk Veterans (33,993 who received care coordination and 292,995 who did not).

### 2.4 Matching

**FIGURE 1:**
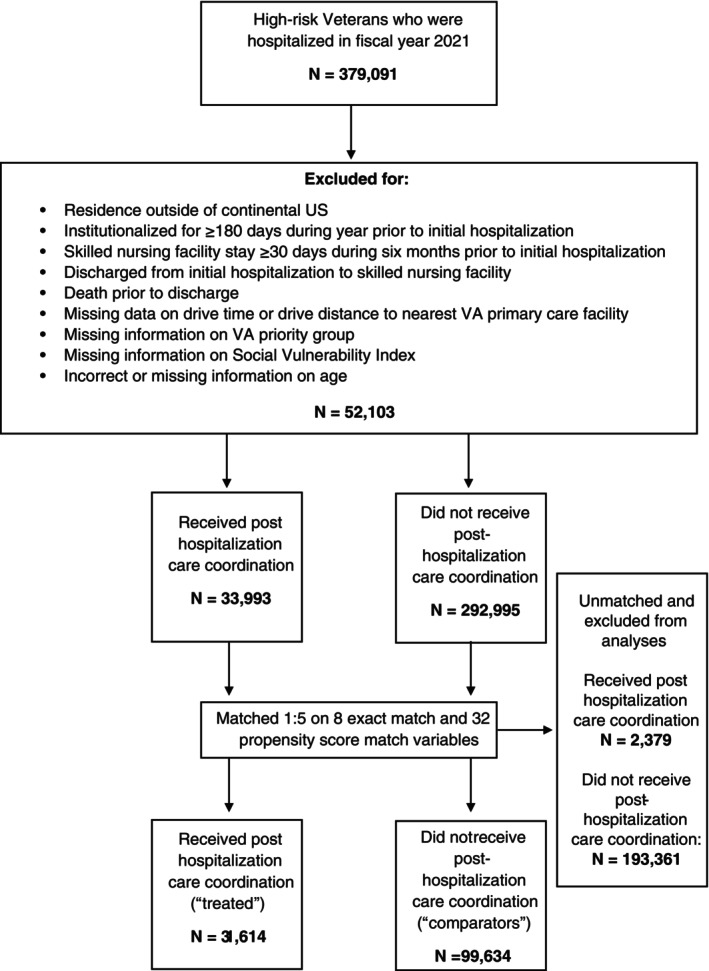
Study flow diagram. Exact match variables: Fiscal year‐quarter of initial hospital discharge; sex; age category; Veterans Integrated Service Network (VISN) of residence; setting of initial hospitalization (VA‐delivered, VA‐purchased, Medicare FFS); Care Coordination and Integrated Case Management early adopter status of the VA facility where the plurality of a Veteran's outpatient care was received during the year prior to initial hospitalization; CAN score quartile based on a Veteran's mean CAN score during the year prior to initial hospitalization; and receipt of care coordination services during the year prior to initial hospitalization. Propensity score match variables: Race; Hispanic/Latino/a/−x ethnicity; insurance status; VA priority group; psychoses diagnosis; depression diagnosis; alcohol use disorder diagnosis; substance use disorder diagnosis; Elixhauser readmission risk score; COVID‐19 infection status during the year prior to initial hospitalization; COVID‐19 vaccination status; VA‐delivered hospitalization count in the year prior to initial hospitalization; Medicare FFS hospitalization count in the year prior to initial hospitalization; VA‐purchased hospitalization count in the year prior to initial hospitalization; total hospitalization count in the year prior to initial hospitalization; VA primary care visit count in the year prior to initial hospitalization; VA mental health care visit count in the year prior to initial hospitalization; VA specialty care visit count in the year prior to initial hospitalization; VA reliance in the year prior to initial hospitalization; urban or rural residence; residence in a primary care health professional shortage area; residence in a mental health care health professional shortage area; drive time in minutes to nearest VA facility; drive distance in miles to nearest VA facility; SVI indicators for socioeconomic status, household composition, racial and ethnic minority status, housing type and transportation; county long‐term bed supply; county hospital bed supply; length of stay of initial hospitalization; primary diagnosis of COVID‐19 for initial hospitalization. Abbreviations: CAN, care assessment of need; FFS, Fee‐for‐service; SVI, Social Vulnerability Index; VA, Veterans Health Administration.

Despite VA care coordination initiatives underway, little is known about how providers determine which high‐risk Veterans should receive care coordination services. However, a recent study identified factors associated with receipt of care coordination services among high‐risk Veterans [28]. With this information, high‐risk Veterans were matched on 40 variables, including eight exact match and 32 propensity score match variables associated with high‐risk Veterans' likelihood of receiving care coordination and which may be associated with Veterans' risk for readmission. Exact match variables included fiscal year‐quarter of initial hospital discharge; sex (male, female); age category (< 45, 45–64, 65–74, 75–84, 85+); Veterans Integrated Service Network (VISN) of residence (VISNs are regional systems of VA care for Veterans in the US, divided into 18 regions); setting of initial hospitalization (VA‐delivered, VA‐purchased, Medicare FFS); Care Coordination and Integrated Case Management (CCICM) early adopter status (yes, no) of the VA facility where the plurality of a Veteran's outpatient care was received during the year prior to initial hospitalization (CCICM is a VA practice framework with the primary focus of improving care coordination, collaboration, communication, and integration at VA facilities; early adopter sites were those that had completed implementation of the CCICM practice framework prior to the study period) [29, 30]; CAN score quartile based on a Veteran's mean CAN score during the year prior to initial hospitalization; and receipt of care coordination services during the year prior to initial hospitalization (yes/no). See Figure 1 footnote for the full set of propensity score matched variables. Propensity scores were estimated based on a model with a 0.1 propensity score caliper. Matching without replacement of treated and comparators was performed on a 1:5 ratio.

### 2.5 Measures

Our main independent variable was a binary indicator for receipt of VA care coordination within 1 day after hospital discharge, which was defined by the presence of a Current Procedural Terminology (CPT)/Healthcare Common Procedure Coding System (HCPCS) code for transitional care management (99495, 99496), care management (99366–99368), chronic care management (G0506, T1016, T1017, 99491), complex chronic care management (99487, 99489, 99490), or behavioral health care management (G0511, G0512, G2214, 99484, 99492–99494) in the VA Corporate Data Warehouse. Only the first instance of post‐hospitalization care coordination was considered; post‐hospitalization care coordination services that were delivered outside of the VA were not considered. Of note, nearly 80% of the care coordination encounters that we identified were documented with code T1016.

Because we were interested in examining the risk of all‐cause and also more specifically ACSC readmissions, two sets of competing risks outcomes were created for the first post‐hospitalization event in the 30 days following the match date. The first outcome was post‐hospitalization all‐cause readmission with death as a competing risk. The second outcome was post‐hospitalization ACSC readmission, with non‐ACSC readmission and death as competing risks. All‐cause readmissions were defined as any VA‐delivered, VA‐purchased, or Medicare FFS hospital readmission to an acute‐care bed unit [31, 32, 33]. ACSC readmissions were defined as all‐cause readmissions that were potentially preventable based on the Agency for Healthcare Research & Quality Prevention Quality Indicators, which are defined based on the principal diagnosis of a hospital stay and have been validated for identifying hospitalizations for ACSCs [34, 35]. Non‐ACSC readmissions were those not considered ACSC based on the Prevention Quality Indicators.

Variables used to describe our cohort included demographic, geographic, VA facility, and clinical characteristics, and health care use variables collected for a baseline period prior to a Veteran's initial hospitalization, as well as characteristics of Veterans' initial hospitalizations. Sociodemographic variables were ascertained in the year prior to initial hospitalization and included sex, age group, VA priority group (a numeric category from 1 to 8 assigned to a Veteran based on their military service history, VA disability rating, income level, Medicaid eligibility, and receipt of other VA benefits), and race and ethnicity (as social constructs and not biological constructs). Race and ethnicity were self‐reported by Veterans and collected in the electronic health record. Although race and ethnicity were not the primary focus of this study, they were included because health care use and mortality are known to vary by race and ethnicity. VA facility and geographic variables were also ascertained in the year prior to initial hospitalization and included whether a VA facility was an early adopter of the CCICM practice framework (practice framework for standardizing and integrating care coordination) [29, 30], rurality of residence, residence in a primary care health professional shortage area, residence in a mental health professional shortage area, drive time and distance to the nearest VA facility, county‐level skilled nursing facility and inpatient bed supply, and four social vulnerability indicators based on the Center for Disease Control and Prevention's Social Vulnerability Index (i.e., socioeconomic status, household characteristics, racial and ethnic minority status, and housing type and transportation) [36, 37]. Baseline clinical and health care use variables were ascertained in the one to 2 years prior to initial hospitalization and included mean CAN score (2 year), insurance status (one year), receipt of care coordination (1 year), alcohol use disorder diagnosis (2 years), depression diagnosis (2 years), substance (other than alcohol) use disorder diagnosis (2 years), psychoses diagnosis (2 years), Elixhauser readmission risk score (2 years), COVID‐19 infection (1 year), COVID‐19 vaccination status (2 year), VA reliance (1 year; a measure of the proportion of a Veterans' outpatient care that is delivered by VA) [38], and count of prior hospitalizations (1 year; VA‐delivered, VA‐purchased, Medicare FFS), VA primary care visits (1 year), VA specialty care visits (1 year), and VA mental health visits (2 year). Characteristics of initial hospitalizations included FY‐quarter of discharge, setting (VA‐delivered, VA‐purchased, Medicare FFS), length of stay, and whether the primary diagnosis for the hospitalization was COVID‐19.

### 2.6 Statistical Analyses

Patient characteristics and matching variables were summarized pre‐ and post‐matching using means and standard deviations for continuous variables and frequencies and percentages for categorical variables, and compared across treated and comparator groups using absolute standard mean differences (SMDs) [39, 40, 41]. Cumulative incidence plots were used to assess overall trends in time to first post‐hospitalization event, including all‐cause readmission, ACSC readmission, and death.

For our primary analysis, we used Fine‐Gray competing risk models [42] to estimate adjusted sub‐hazard ratios (aSHRs) and 95% confidence intervals (CIs) for each post‐hospitalization event among the matched cohort. Two separate models were estimated: one for all‐cause readmission and death, and another for ACSC readmission, non‐ACSC readmission, and death. Covariates with SMDs > 0.1 were used as adjustment variables in regression models. These included receipt of care coordination during the year prior to initial hospitalization (SMD 0.13), setting of initial hospitalization (SMD 0.11), count of mental health visits in the year prior to initial hospitalization (SMD −0.15), length of stay of the initial hospitalization (SMD 0.14), and state (SMD 0.32) and Veterans Integrated Service Network of Veteran residence (SMD 0.27). In accordance with a per‐protocol approach [43], assigned untreated Veterans who later received care coordination (i.e., untreated Veterans who experienced cross‐over to treatment during follow‐up) were censored, and outcomes that occurred among these Veterans after censoring were not analyzed.

Four sensitivity analyses were performed to evaluate the robustness of our results. First, we performed inverse probability of censoring weights [44], described in the Supplement, to account for censoring due to cross‐over to treatment among comparators during 30‐day follow‐up. Second, we performed an analysis with an enhanced set of exact match variables about initial hospitalizations. These included whether the initial hospitalization involved an intensive care unit (ICU) stay, quartiles of Diagnostic Related Group (DRG) weights [45], and whether the initial hospital discharge occurred on a weekday or weekend. Third, because we did not have Medicare Advantage encounter data, we performed an analysis that excluded treated Veterans and their matched comparators, as well as individual comparators who had Medicare Advantage coverage. Fourth, and in an attempt to further address endogeneity between receipt of care coordination and readmission, we performed an analysis that was limited to treated and matched comparators who had the same clinical indication for their initial admission, COVID‐19. We chose COVID‐19 because it was the most common reason for initial admission among our analytic cohort.

Additionally, two sets of exploratory subgroup analyses were performed using the same methods as described for our primary analysis. The first subgroup analysis grouped Veterans by the setting of their initial hospitalization: VA‐delivered, VA‐purchased, or Medicare FFS. The second analysis grouped Veterans by the CCICM early adopter status (i.e., early adopter, non‐early adopter) of the VA facility at which they received the plurality of their outpatient care in the year prior to their initial hospitalization. Both the setting of initial hospitalization and CCICM early adopter status were exact‐match variables, resulting in balanced matches across treatment status in all subgroups.

Matching was performed using the PSMATCH procedure from SAS/STAT version 15.1 base SAS version 9.4_M6; all other analyses were performed using R version 4.4.1.

## 3 Results

### 3.1 Matching

A total of 326,988 high‐risk Veterans met our study inclusion criteria (Figure 1). Prior to matching, there were medium to large differences in characteristics between treated and comparators (Table S1).

Overall, 93% of treated Veterans were matched with at least one comparator, with 34% matched with five comparators. Matching resulted in an analytic sample of 131,248 Veterans who were discharged alive and matched, of whom 24% (*n* = 31,614) received care coordination on the day of or the first day after discharge (Figure 1, Table 1, Table S1).

**TABLE 1:** Participant characteristics by post‐hospitalization care coordination status.

			Post‐hospitalization care coordination status				
Characteristic	Overall (*N* or mean) *N* = 131,248	Overall (% or SD) 100%	No (*N* or mean) *N* = 99,634	No (% or SD) 76%	Yes (*N* or mean) *N* = 31,614	Yes (% or SD) 24%	SMD
Sex, no. (%)							0.05
Female	4519	3.4	3198	3.2	1321	4.2	
Male	126,729	96.6	96,436	96.8	30,293	95.8	
Age group (years), no. (%)							0.06
< 45	3757	2.9	2698	2.7	1059	3.4	
45–64	33,681	25.7	25,587	25.7	8094	25.6	
65–74	52,477	40.0	40,148	40.3	12,329	39.0	
75–84	27,449	20.9	20,936	21.0	6513	20.6	
85+	13,884	10.6	10,265	10.3	3619	11.5	
Mean CAN score category 1‐year pre‐hospitalization, no. (%)							0.06
(3, < 81.6)	27,498	21.0	21,266	21.3	6232	19.7	
(81.6, < 88.3)	27,550	21.0	21,131	21.2	6419	20.3	
(88.3, < 94.5)	33,061	25.2	25,106	25.2	7955	25.2	
(94.5, 99)	43,139	32.9	32,131	32.3	11,008	34.8	
Veteran's assigned VA facility is a CCICM early adopter site, No. (%)	26,040	19.8	19,714	19.8	6326	20.0	0.01
Receipt of care coordination 1‐year pre‐hospitalization, no. (%)	59,788	45.6	43,829	44.0	15,959	50.5	0.13
Discharge quarter, no. (%)							0.01
Q1 FY21	44,275	33.7	33,658	33.8	10,617	33.6	
Q2 FY21	33,027	25.2	25,056	25.2	7971	25.2	
Q3 FY21	30,475	23.2	23,037	23.1	7438	23.5	
Q4 FY21	23,471	17.9	17,883	18.0	5588	17.7	
Setting of initial hospitalization, no. (%)							0.11
Medicare FFS	2665	2.0	2159	2.2	506	1.6	
VA‐purchased	11,517	8.8	9406	9.4	2111	6.7	
VA‐delivered	117,066	89.2	88,069	88.4	28,997	91.7	
Race, no. (%)							0.04
American Indian/Alaska Native	991	0.8	752	0.8	239	0.8	
Asian	468	0.4	372	0.4	96	0.3	
Black/African American	34,445	26.2	25,812	25.9	8633	27.3	
Multiracial	1137	0.9	850	0.9	287	0.9	
Native Hawaiian/Pacific Islander	859	0.7	678	0.7	181	0.6	
White	91,791	69.9	69,968	70.2	21,823	69.0	
Other/unknown race identity	1557	1.2	1202	1.2	355	1.1	
Hispanic/latino/a/−x ethnicity, no. (%)							0.02
Non‐Hispanic/latino/a/−x	124,617	95.0	94,488	94.8	30,129	95.3	
Hispanic/latino/a/−x	6631	5.1	5146	5.2	1485	4.7	
Insurance/coverage status, no. (%)							0.05
VA only	16,328	12.4	12,373	12.4	3955	12.5	
VA and Medicare	97,818	74.5	74,695	75.0	23,123	73.1	
VA and multiple	17,102	13.0	12,566	12.6	4536	14.4	
VA priority group, no. (%)							0.05
Group 1	54,649	41.6	41,821	42.0	12,828	40.6	
Group 2	6817	5.2	5181	5.2	1636	5.2	
Group 3	12,322	9.4	9441	9.5	2881	9.1	
Group 4	4676	3.6	3414	3.4	1262	4.0	
Group 5	38,772	29.5	29,020	29.1	9752	30.9	
Group 6	1579	1.2	1215	1.2	364	1.2	
Group 7	3510	2.7	2652	2.7	858	2.7	
Group 8	8923	6.8	6890	6.9	2033	6.4	
Psychoses, no. (%)	27,645	21.1	20,333	20.4	7312	23.1	0.07
Depression, no. (%)	58,821	44.8	44,151	44.3	14,670	46.4	0.04
Alcohol use disorder, no. (%)	32,497	24.8	24,150	24.2	8347	26.4	0.05
Substance use disorder, no. (%)	23,126	17.6	17,024	17.1	6102	19.3	0.06
Elixhauser score, readmission, mean (SD)	43.64	26.2	43.4	26.1	44.42	26.5	0.04
COVID‐19 infection 1‐year pre‐hospitalization, no. (%)	9395	7.2	7144	7.2	2251	7.1	0.00
COVID‐19 vaccination status, no. (%)							0.02
Fully vaccinated	42,602	32.5	32,597	32.7	10,005	31.7	
Not fully vaccinated	88,646	67.5	67,037	67.3	21,609	68.4	
VA hospitalization count 1‐year pre‐hospitalization, mean (SD)	0.5	1.0	0.5	1.0	0.5	1.1	0.03
Medicare FFS hospitalization count 1‐year pre‐hospitalization, mean (SD)	0.1	0.3	0.1	0.3	0.1	0.3	< 0.01
VA‐purchased hospitalization count 1‐year pre‐hospitalization, mean (SD)	0.1	0.5	0.1	0.5	0.1	0.5	0.01
All setting hospitalization count 1‐year pre‐hospitalization, mean (SD)	0.6	1.3	0.6	1.3	0.7	1.3	0.02
VA primary care visit count 1‐year pre‐hospitalization, mean (SD)	11.3	9.2	11.3	9.0	11.3	9.7	< 0.01
VA mental health care visit count 1‐year pre‐hospitalization, mean (SD)	8.3	19.9	7.5	18.3	10.8	23.9	0.15
VA specialty care visit count 1‐year pre‐hospitalization, mean (SD)	25.8	17.3	25.7	17.0	26.0	18.2	0.01
VA reliance, mean (SD)	0.9	0.2	0.9	0.2	0.9	0.2	0.01
Urban or rural residence, no. (%)							0.03
Urban	94,859	72.3	71,699	72.0	23,160	73.3	
Rural/highly rural	36,389	27.7	27,935	28.0	8454	26.7	
Residence in PC‐HPSA, no. (%)	34,378	26.2	26,737	26.8	7641	24.2	0.06
Residence in MH‐HPSA, no. (%)	41,961	32.0	32,955	33.1	9006	28.5	0.10
Drive time to nearest VA (minutes), no. (%)							0.04
0–10	40,337	30.7	30,290	30.4	10,047	31.8	
11–20	49,822	38.0	37,773	37.9	12,049	38.1	
21–30	21,589	16.5	16,542	16.6	5047	16.0	
> 30	19,500	14.9	15,029	15.1	4471	14.1	
Drive distance to VA (miles), no. (%)							0.05
0–5	38,715	29.5	28,960	29.1	9755	30.9	
6–10	33,205	25.3	25,144	25.2	8061	25.5	
11–20	32,833	25.0	25,025	25.1	7808	24.7	
21–40	20,561	15.7	15,899	16.0	4662	14.8	
> 40	5934	4.52	4606	4.6	1328	4.2	
SVI, socioeconomic status, mean (SD)	0.6	0.3	0.6	0.3	0.6	0.3	0.06
SVI, household characteristics, mean (SD)	0.5	0.3	0.5	0.3	0.5	0.3	0.04
SVI, racial and ethnic minority status, mean (SD)	0.7	0.3	0.7	0.2	0.7	0.3	0.03
SVI, housing type and transportation, mean (SD)	0.6	0.2	0.6	0.2	0.6	0.2	0.01
County long‐term care bed count, mean (SD)	4740.6	7913.7	4756.7	7999.3	4689.8	7637.5	0.01
County hospital bed count, mean (SD)	2841.5	4367.8	2853.1	4370.9	2805.2	4357.9	0.01
Length of initial hospitalization (days), no. (%)							0.14
1	31,907	24.3	24,844	24.9	7063	22.3	
2	24,190	18.4	18,839	18.9	5351	16.9	
3–7	53,622	40.9	40,790	40.9	12,832	40.6	
8–14	14,902	11.4	10,671	10.7	4231	13.4	
15+	6627	5.1	4490	4.5	2137	6.8	
Primary diagnosis of COVID‐19 for initial hospitalization, no. (%)	9988	7.6	7596	7.6	2392	7.6	< 0.01

*Note:* Veterans Integrated Service Network and state of residence omitted for table brevity.

Abbreviations: CAN, care assessment of need; CCICM, Care Coordination and Integrated Case Management; CPT, Current Procedural Terminology; MH‐HPSA, mental health care health professional shortage area; PC‐HPSA, primary care health professional shortage area; SMD, absolute standardized mean difference; SVI, Social Vulnerability index; VA, veterans health administration.

### 3.2 Matched Participant Characteristics

Following matching, most characteristics were well‐balanced between treated and comparator groups (Table 1). For example, high‐risk Veterans were predominantly male (95.8% vs. 96.8%), non‐Hispanic/Latino/a/‐x ethnicity (95.3% vs. 94.8%), ≥ 65 years of age (69.52% vs. 69.95%), and had Medicare coverage in addition to their VA benefits (71.1% vs. 71.6%). Most resided in urban areas (73.3% vs. 72.0%) and had a ≤ 30‐min drive time from their residence to the nearest VA facility (85.9% vs. 84.9%). Regarding initial admissions, most occurred within VA facilities (91.7% vs. 88.4%). Regarding clinical characteristics, nearly half of high‐risk Veterans had a diagnosis of depression (46.4% vs. 44.3%), about one quarter had diagnoses of psychoses (23.1% vs. 20.4%) and alcohol use disorder (26.4% vs. 24.2%), and one in five had a diagnosis of substance use disorder (19.3% vs. 17.1%). Although differences were small, high‐risk Veterans who received post‐hospitalization care coordination were more likely to have received care coordination in the year prior to their initial hospitalization (50.5% vs. 44.0%, SMD 0.13) and to have had initial hospitalizations in VA facilities (91.7% vs. 88.4%, SMD 0.11); there were also small differences in initial hospitalization length of stay (SMD 0.14). Although high‐risk Veterans were balanced in their psychiatric and substance use diagnoses, those who received post‐hospitalization care coordination had somewhat greater mean count of VA mental health visits in the year prior to initial hospitalization than matched comparators (10.8, SD 23.9 visits vs. 7.5, SD 18.3 visits, SMD 0.15). In addition, state (SMD 0.32) and Veterans Integrated Service Network (SDM 0.27) of residence varied somewhat across treated and comparator groups.

### 3.3 Incidence of Hospital Readmissions by Post‐Hospitalization Care Coordination Status

**FIGURE 2:**
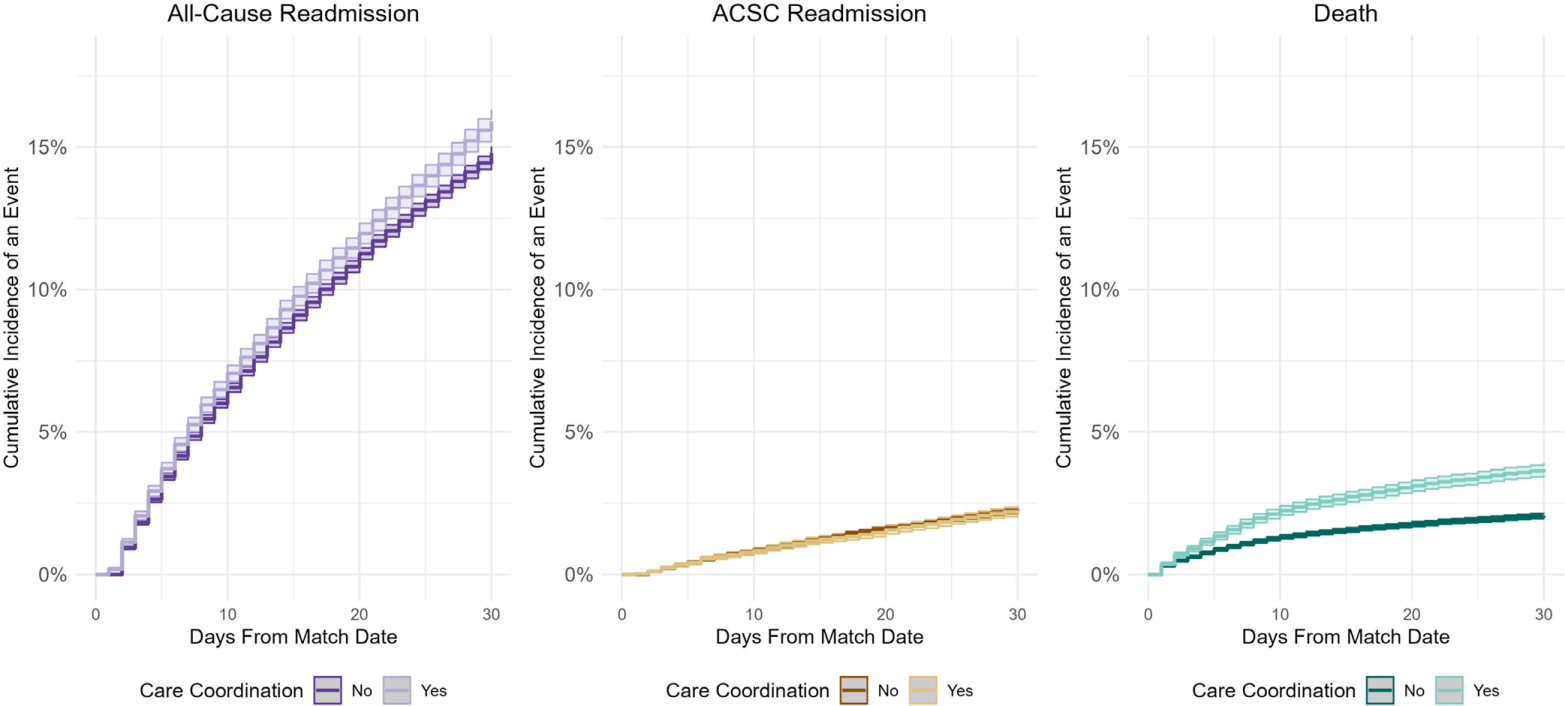
Cumulative incidence of 30‐day hospital readmissions and death by post‐hospitalization care coordination status. Abbreviations: ACSC, ambulatory care‐sensitive condition.

The cumulative incidence of 30‐day all‐cause hospital readmission was greater among treated Veterans than among untreated matched comparators (15.9% vs. 13.5%), with a cumulative incidence difference of 2.36 percentage points (95% CI 1.91, 2.82) (Figure 2, Panel A, Table 2). The cumulative incidence of 30‐day ACSC readmission was similar among treated and comparators (2.3% vs. 2.1%), with a cumulative incidence difference of 0.17 percentage points (95% CI –0.36, 0.02) (Figure 2, Panel B, Table 2). After accounting for competing risks and adjusting for variables that remained imbalanced across groups after matching, receipt of post‐hospitalization care coordination was associated with no difference in all‐cause (aSHR 1.03, 95% CI 1.00, 1.07) or ACSC (aSHR 0.97, 95% CI 0.89, 1.05) readmission (Table 2), while the competing risk of death was greater among treated Veterans than among comparators (aSHR 1.85, 95% CI 1.72, 2.00) (Table 2).

**TABLE 2:** Risk of 30‐day hospital readmissions and death by post‐hospitalization care coordination status.

Outcome	Cumulative incidence						
	Overall	Post‐hospitalization care coordination status		Unadjusted percentage point difference		Adjusted subhazard ratio (aSHR)	
	*N* = 131,248	Yes *N* = 31,614 (24%)	No *N* = 99,634 (76%)	Estimate	95% CI	Estimate	95% CI
All‐cause readmission^a^	14.1%	15.9%	13.5%	2.36	1.91, 2.82	1.03	1.00, 1.07
ACSC readmission^b^	2.1%	2.3%	2.1%	0.17	−0.02, 0.36	0.97	0.89, 1.05
Death^c^	2.4%	3.7%	1.9%	1.76	1.53, 1.98	1.85	1.72, 2.00

*Note:* All models were adjusted by matching variables with SMDs > 0.1.

Abbreviations: ACSC, ambulatory care‐sensitive condition; aSHR, adjusted sub‐hazard ratio; CI, confidence interval.

^a^ All‐cause readmission aSHR and 95% CI estimated in a model with death as a competing risk.

^b^ ACSC readmission aSHR and 95% CI estimated in a model with non‐ACSC readmission and death as competing risks.

^c^ Death aSHR and 95% CI are the same in both models.

### 3.4 Sensitivity Results

After implementing inverse probability of censoring weights to account for cross‐over among comparators and censoring of their outcomes (Table S2), and in a separate sensitivity analysis applying Medicare Advantage exclusions (Table S3), receipt of post‐hospitalization care coordination was associated with a slightly greater risk of all‐cause readmission and no difference in the risk of ACSC readmission. After including an enhanced set of matching variables (Table S4), and in a separate sensitivity analysis limited to treated and matched comparators who had the same clinical indication for their initial admission, COVID‐19 (Table S5), there were no significant differences in the risk of all‐cause or ACSC readmission. In all sensitivity analyses except for the COVID‐19 analysis, receipt of post‐hospitalization care coordination was associated with an increased (competing) risk of death (Tables S2–S5).

### 3.5 Post Hoc Subgroup Results

Subgroup results based on the setting of the initial hospitalization were mostly similar to our primary results for all‐cause readmission but varied for ACSC readmission. For example, among Veterans whose initial hospitalizations occurred in VA facilities, receipt of post‐hospitalization care coordination was associated with a somewhat greater risk of all‐cause readmission (aSHR 1.04, 95% CI 1.01, 1.08) and no difference in ACSC readmission (aSHR 0.99, 95% CI 0.91, 1.09) (Table 3). In VA‐purchased and Medicare FFS settings, adjusted subhazard ratios were not statistically significantly different from 1.00 for all‐cause or ACSC readmission (Table 3). However, in CCICM subgroup analyses, Veterans who received the plurality of their outpatient care at VA facilities that were early adopters of the CCICM practice framework had a lower risk of ACSC readmission (0.81, 95% CI 0.67, 0.99) (Table 3). There was no difference in the risk of all‐cause readmission between treated and comparators at CCICM early adopter facilities (aSHR 1.01, 95% CI 0.94, 1.09) (Table 3). Among all subgroups we examined and similar to our primary analyses, receipt of post‐hospitalization care coordination was associated with a greater (competing) risk of death at 30 days (Table 3).

**TABLE 3:** Comparisons of 30‐day hospital readmissions and death among treated and comparators, by setting of initial hospitalization and CCICM early adopter status of the VA facility where Veteran received the plurality of their outpatient care in 1 year prior to initial hospitalization.

Subgroup & Outcome	Cumulative incidence						
	Overall	Post‐hospitalization care coordination status		Unadjusted percentage point difference		Adjusted subhazard ratio (aSHR)	
VA‐delivered	*N* = 117,066	Yes	No	Estimate	95% CI	Estimate	95% CI
		*N* = 28,997 (25%)	*N* = 88,069 (75%)				
All‐cause readmission^a^	13.8%	15.8%	13.2%	2.58	2.10, 3.06	1.04	1.01, 1.08
ACSC readmission^b^	2.1%	2.3%	2.0%	0.29	0.09, 0.48	0.99	0.91, 1.09
Death^c^	2.2%	3.6%	1.8%	1.89	1.65, 2.12	1.90	1.75, 2.07

VA‐purchased	*N* = 11,517	Yes	No	Estimate	95% CI	Estimate	95% CI
		*N* = 2111 (18%)	*N* = 9406 (82%)				
All‐cause readmission^a^	16.0%	17.6%	15.6%	1.97	0.16, 3.78	0.95	0.85, 1.08
ACSC readmission^b^	2.3%	1.6%	2.5%	−0.85	−1.50, −0.20	0.70	0.48, 1.02
Death^c^	2.9%	3.3%	2.8%	0.44	−0.42, 1.30	1.61	1.23, 2.12

Medicare FFS	*N* = 2665	Yes	No	Estimate	95% CI	Estimate	95% CI
		*N* = 506 (19%)	*N* = 2159 (81%)				
All‐cause readmission^a^	18.3%	16.6%	18.7%	−2.11	−5.87, 1.65	0.81	0.63, 1.03
ACSC readmission^b^	3.0%	2.2%	3.2%	−1.07	−2.66, 0.53	0.81	0.42, 1.54
Death^c^	6.5%	9.1%	5.9%	3.16	0.35, 5.98	1.46	1.03, 2.09

CCICM early adopter	*N* = 26,040	Yes	No	Estimate	95% CI	Estimate	95% CI
		*N* = 6326 (24%)	*N* = 19,714 (76%)				
All‐cause readmission^a^	14.4%	16.2%	13.8%	2.39	1.35, 3.43	1.01	0.94, 1.09
ACSC readmission^b^	2.3%	2.2%	2.3%	−0.12	−0.55, 0.30	0.81	0.67, 0.99
Death^c^	2.5%	4.4%	1.9%	2.49	1.94, 3.04	2.19	1.85, 2.59

Non‐CCICM early adopter	*N* = 105,208	Yes	No	Estimate	95% CI	Estimate	95% CI
		*N* = 25,288 (24%)	*N* = 79,920 (76%)				
All‐cause readmission^a^	14.1%	15.9%	13.5%	2.36	1.85, 2.87	1.04	1.00, 1.07
ACSC readmission^b^	2.1%	2.3%	2.0%	0.24	0.03, 0.45	1.01	0.92, 1.11
Death^c^	2.3%	3.5%	2.0%	1.57	1.32, 1.82	1.80	1.65, 1.96

*Note:* Footnote: All models were adjusted by matching variables with SMDs > 0.1.

Abbreviations: ACSC, ambulatory care‐sensitive condition; aSHR, adjusted sub‐hazard ratio; CCICM, Care Coordination and Integrated Case Management; CI, confidence interval; FFS, fee for service.

^a^ All‐cause readmission aSHR and 95% CI estimated in a model with death as a competing risk.

^b^ ACSC readmission aSHR and 95% CI estimated in a model with non‐ACSC readmission and death as competing risks.

^c^ Death aSHR and 95% CI are the same in both models.

## 4 Discussion

Our analysis of a well‐matched cohort of high‐risk Veterans demonstrated that receipt of care coordination within 1 day after hospital discharge was associated with no difference in the risk of 30‐day all‐cause hospital readmission in primary and subgroup analyses, and either no difference or a small increased risk for readmission in sensitivity analyses. Regarding ACSC (i.e., potentially preventable) readmission, in primary and sensitivity analyses we also observed no difference in readmission risk between high‐risk Veterans who did and did not receive post‐hospitalization care coordination. However, we observed a significantly lower risk of ACSC readmission among the subgroup of Veterans who received post‐hospitalization care coordination and the plurality of their outpatient care at VA facilities that were early adopters of CCICM—a VA practice framework for standardizing and delivering enhanced care coordination to high‐risk Veterans.

Our null findings parallel certain existing literature. For example, in a meta‐analysis, receipt of brief post‐discharge contacts (e.g., phone calls) within 7 days of hospital discharge was associated with no difference in 30‐day readmissions [10], a finding supported by systematic reviews [10, 46, 47]. In addition, an early VA trial that assigned Veterans to a case manager at hospital discharge had no effect on readmissions [48], and a recent VA initiative to provide longitudinal care coordination to Veterans who use both VA and non‐VA hospitals was associated with no difference in readmissions [49]. On the other hand, a seminal randomized controlled trial conducted within the VA found that enhanced access to primary care shortly after hospital discharge was associated with increased readmissions which were thought to stem in part from providers identifying new/continuing needs for hospital care during post‐discharge visits [11]. This reasoning could explain the positive association we observed in certain analyses between receipt of post‐hospitalization care coordination and all‐cause readmission. However, inconsistencies between our primary and sensitivity analysis and the small magnitude of association should be considered when interpreting these results.

Our finding that treated Veterans had a lower risk of ACSC readmission than comparators if they received the plurality of their outpatient care at VA facilities that were early adopters of CCICM warrants further study. Prior to CCICM adoption, Veterans could have multiple coordinators who arranged different aspects of their care and who may or may not have communicated with each other—a strategy which some suggest led to siloing and fragmentation [50]. First launched in 2016 in partnership between the VA Offices of Nursing and Social Work and Nursing Services, CCICM brings together disparate care management services and providers under one framework with defined standards of practice that support delivery of care coordination, care management, and case management services, and involves assigning one Lead Coordinator to Veterans who need moderate to complex care coordination, with the goal of standardizing care coordination as a service across the VA system [51]. Program data indicate that Veterans enrolled in CCICM have 79% fewer hospitalizations and ED visits, and in 2025, CCICM implementation metrics were integrated into VA performance standards nationally [52]. Care coordination provided at CCICM early adopter VA facilities may have been different (e.g., more comprehensive) than that provided at other VA facilities. However, formal evaluations of CCICM are ongoing; whether CCICM adoption is causally linked with reduced readmissions among high‐risk Veterans requires further study.

Although it is difficult to identify the characteristics of strategies that lead to positive hospital‐to‐home transitions [53], there is some evidence that successful approaches address more components of the care transition, include mechanisms to assess and respond to individuals' peri‐discharge needs, and bridge pre‐ and post‐discharge settings [53, 54, 55, 56]. Supporting this, a recent VA study found that VA facilities that use a greater number of recommended transitional care management processes (e.g., use of a care transitions case manager; printed follow‐up instructions at discharge; post‐hospitalization follow‐up appointments made prior to discharge) have lower hospital readmission rates [57]. Nevertheless, care coordination interventions described in the literature, as well as those delivered across VA, are heterogeneous in their components and intensity [48, 54, 55, 56]. And, despite the array of CPT/HCPCS codes available for documenting care coordination, more than 80% of care coordination encounters among high‐risk Veterans are captured via HCPCS code T1016 [28]—a generic case management code. Further, CPT/HCPCS codes, even when used with specificity, do not detail the specific activities carried out during care coordination encounters. Given that both the intensity and components of care coordination can influence its effectiveness, efforts to enhance care coordination measurement and data are needed to more fully evaluate its impact on patient outcomes such as hospital readmissions, particularly for observational studies that rely on extant administrative data.

### 4.1 Limitations

This study has limitations. First and foremost, despite developing a well‐balanced cohort of Veterans who did and did not receive post‐hospitalization care coordination services based on an extensive list of factors that have been shown to be associated with receipt of care coordination among high‐risk Veterans [28], residual confounding may remain, and results should not be interpreted as causal. For example, care teams, family, and caregivers may have knowledge about Veterans that is not captured in extant data but may influence both receipt of care coordination and readmissions, such as information about social risks and needs, and the extent of informal care coordination from family and friends. In addition, Veterans may receive referrals for care coordination from family or caregivers and can refer themselves for care coordination. These referrals are not captured in extant VA data and may be related to unobserved factors associated with risks of readmissions and/or death. That we observed an increased (competing) risk of death among Veterans who received post‐hospitalization care coordination suggests such residual confounding may exist. Nonetheless, we believe these findings are useful for understanding the post‐hospitalization care coordination context and an important step in examining effectiveness. Next, we only considered the first instance of post‐hospitalization care coordination in treatment assignment. Although most Veterans (> 80%, data not shown) in our study had only one instance of post‐hospitalization care coordination, it is possible that the relationship between care coordination and hospital readmission may look different if more services are delivered and considered. Third, the sensitivity and specificity of CPT/HCPCS codes for identifying receipt of VA care coordination is unknown; it is possible that some Veterans received care coordination that was not documented by an included CPT/HCPCS code and were thus misclassified as untreated, which could bias our results. Last, this study was conducted among high‐risk Veterans who received care within the VA, who were predominantly male sex, non‐Hispanic/Latino/a/−x ethnicity, and with high levels of mental health comorbidity. Therefore, the generalizability of our findings to populations with different risk profiles may be affected.

## 5 Conclusion

In a retrospective cohort of matched high‐risk Veterans, we found that receipt of post‐hospitalization care coordination was associated with small or no differences in the risk of 30‐day all‐cause and ACSC readmissions. Care coordination services, including those delivered within the VA and soon after hospital discharge, can be varied and complex, and the effects of care coordination inputs are challenging to unpack using extant data. Efforts to enhance care coordination data and measurement, and future research to explore effective components or sets of components of post‐hospitalization care coordination are needed, as is identification of effective hospital‐to‐home care transition models for high‐risk Veterans.

## Conflicts of Interest

The authors declare no conflicts of interest.

## Supporting information


**Data S1:** hesr70044‐sup‐0001‐TableS1.docx.

## Data Availability

The data that support the findings of this study may be available on request from the corresponding author. The data are not publicly available due to privacy or ethical restrictions.

## References

[1] Agency for Healthcare Research & Quality , “Hospital Admission Versus Readmission Costs,” 2020.

[2] US Department of Health and Human Services , “Medicare Program; Revisions to Payment Policies Under the Physician Fee Schedule, DME Face‐To‐Face Encounters, Elimination of the Requirement for Termination of Non‐Random Prepayment Complex Medical Review and Other Revisions to Part B for CY 2013. Final Rule With Comment Period,” 2012. 23155552

[3] L. M. Marcotte , A. Reddy , L. Zhou , S. C. Miller , C. Hudelson , and J. M. Liao , “Trends in Utilization of Transitional Care Management in the United States,” JAMA Network Open 3, no. 1 (2020): e1919571, 10.1001/jamanetworkopen.2019.19571. 31968111 PMC6991271

[4] T. S. Anderson , S. J. Herzig , E. R. Marcantonio , R. W. Yeh , J. Souza , and B. E. Landon , “Medicare Transitional Care Management Program and Changes in Timely Postdischarge Follow‐Up,” JAMA Health Forum 5, no. 4 (2024): e240417, 10.1001/jamahealthforum.2024.0417. 38607641 PMC11065163

[5] A. B. Bindman and D. F. Cox , “Changes in Health Care Costs and Mortality Associated With Transitional Care Management Services After a Discharge Among Medicare Beneficiaries,” JAMA Internal Medicine 178, no. 9 (2018): 1165–1171, 10.1001/jamainternmed.2018.2572. 30073240 PMC6583218

[6] P. Boersma , R. Cohen , C. Zelaya , and E. Moy , “Multiple Chronic Conditions Among Veterans and Nonveterans: United States,” Centers for Disease Control and Prevention 13 (2021): 1–12.

[7] M. Olenick , M. Flowers , and V. J. Diaz , “US Veterans and Their Unique Issues: Enhancing Health Care Professional Awareness,” Advances in Medical Education and Practice 6 (2015): 635–639, 10.2147/AMEP.S89479. 26664252 PMC4671760

[8] K. Carey and T. Stefos , “The Cost of Hospital Readmissions: Evidence From the VA,” Health Care Management Science 19, no. 3 (2016): 241–248, 10.1007/s10729-014-9316-9. 25576391

[9] A. M. Kilbourne , D. Hynes , T. O'Toole , and D. Atkins , “A Research Agenda for Care Coordination for Chronic Conditions: Aligning Implementation, Technology, and Policy Strategies,” Translational Behavioral Medicine 8, no. 3 (2018): 515–521, 10.1093/tbm/ibx084. 29800409

[10] J. C. Boggan , S. Sankineni , A. M. Gordon , et al., “Effectiveness of Post‐Discharge Contacts on Health Care Utilization and Patient Satisfaction: A Systematic Review,” 2024. 40138509

[11] M. Weinberger , E. Z. Oddone , and W. G. Henderson , “Does Increased Access to Primary Care Reduce Hospital Readmissions?,” New England Journal of Medicine 334, no. 22 (1996): 1441–1447, 10.1056/NEJM199605303342206. 8618584

[12] D. M. Smith , A. Giobbie‐Hurder , M. Weinberger , et al., “Predicting Non‐Elective Hospital Readmissions: A Multi‐Site Study,” Journal of Clinical Epidemiology 53, no. 11 (2000): 1113–1118, 10.1016/s0895-4356(00)00236-5. 11106884

[13] K. M. Nelson , C. Helfrich , H. Sun , et al., “Implementation of the Patient‐Centered Medical Home in the Veterans Health Administration: Associations With Patient Satisfaction, Quality of Care, Staff Burnout, and Hospital and Emergency Department Use,” JAMA Internal Medicine 174, no. 8 (2014): 1350–1358, 10.1001/jamainternmed.2014.2488. 25055197

[14] E. T. Chang , A. Huynh , C. Yoo , et al., “Impact of Referring High‐Risk Patients to Intensive Outpatient Primary Care Services: A Propensity Score‐Matched Analysis,” Journal of General Internal Medicine 40 (2024): 637–646, 10.1007/s11606-024-08923-3. 39075268 PMC11861449

[15] D. A. Asfaw , M. E. Price , K. M. Carvalho , S. D. Pizer , and M. M. Garrido , “The Effects of the Veterans Health Administration's Referral Coordination Initiative on Referral Patterns and Waiting Times for Specialty Care,” Health Services Research 59, no. 3 (2024): 3, 10.1111/1475-6773.14303. PMC11063088 38553984

[16] L. A. Garvin , M. Pugatch , D. Gurewich , J. N. Pendergast , and C. J. Miller , “Interorganizational Care Coordination of Rural Veterans by Veterans Affairs and Community Care Programs,” Medical Care 59 (2021): S259–S269, 10.1097/MLR.0000000000001542. 33976075 PMC8132902

[17] J. S. Holtrop , Z. Luo , and L. Alexanders , “Inadequate Reimbursement for Care Management to Primary Care Offices,” Journal of American Board of Family Medicine 28, no. 2 (2015): 271–279, 10.3122/jabfm.2015.02.140207. 25748769

[18] C. Boult , A. F. Green , L. B. Boult , J. T. Pacala , C. Snyder , and B. Leff , “Successful Models of Comprehensive Care for Older Adults With Chronic Conditions: Evidence for the Institute of Medicine's “Retooling for an Aging America” Report,” Journal of the American Geriatrics Society 57, no. 12 (2009): 2328–2337, 10.1111/j.1532-5415.2009.02571.x. 20121991

[19] S. R. Counsell , C. M. Callahan , A. B. Buttar , D. O. Clark , and K. I. Frank , “Geriatric Resources for Assessment and Care of Elders (GRACE): A New Model of Primary Care for Low‐Income Seniors,” Journal of the American Geriatrics Society 54, no. 7 (2006): 1136–1141, 10.1111/j.1532-5415.2006.00791.x. 16866688

[20] D. M. Zulman , E. T. Chang , A. Wong , et al., “Effects of Intensive Primary Care on High‐Need Patient Experiences: Survey Findings From a Veterans Affairs Randomized Quality Improvement Trial,” Journal of General Internal Medicine 34, no. Suppl 1 (2019): 75–81, 10.1007/s11606-019-04965-0. PMC6542922 31098977

[21] D. M. Hynes , M. Fischer , M. Fitzgibbon , et al., “Integrating a Medical Home in an Outpatient Dialysis Setting: Effects on Health‐Related Quality of Life,” Journal of General Internal Medicine 34, no. 10 (2019): 2130–2140, 10.1007/s11606-019-05154-9. 31342329 PMC6816601

[22] J. Yoon , E. Chang , L. V. Rubenstein , et al., “Impact of Primary Care Intensive Management on High‐Risk Veterans' Costs and Utilization: A Randomized Quality Improvement Trial,” Annals of Internal Medicine 168, no. 12 (2018): 846–854, 10.7326/M17-3039. 29868706

[23] T. Bodenheimer , “Coordinating Care — A Perilous Journey Through the Health Care System,” New England Journal of Medicine 358, no. 10 (2008): 1064–1071, 10.1056/NEJMhpr0706165. 18322289

[24] M. S. Bauer , C. J. Miller , B. Kim , et al., “Effectiveness of Implementing a Collaborative Chronic Care Model for Clinician Teams on Patient Outcomes and Health Status in Mental Health: A Randomized Clinical Trial,” JAMA Network Open 2, no. 3 (2019): e190230, 10.1001/jamanetworkopen.2019.0230. 30821830 PMC6484628

[25] D. M. Hynes , K. Koelling , K. Stroupe , et al., “Veterans' Access to and Use of Medicare and Veterans Affairs Health Care,” Medical Care 45, no. 3 (2007): 214–223, 10.1097/01.mlr.0000244657.90074.b7. 17304078

[26] D. M. Hynes , M. L. Maciejewski , and D. Atkins , “HSR Commentary: Linking VA and Non‐VA Data to Address Important US Veteran Health Services Research Issues,” Health Services Research 53, no. 3 (2018): 5133–5139, 10.1111/1475-6773.13081. 30430570 PMC6235822

[27] L. Wang , B. Porter , C. Maynard , et al., “Predicting Risk of Hospitalization or Death Among Patients Receiving Primary Care in the Veterans Health Administration,” Medical Care 51 (2013): 368–373, 10.1097/MLR.0b013e31827da95a. 23269113

[28] D. J. Govier , A. Hickok , M. Niederhausen , et al., “Intensity, Characteristics, and Factors Associated With Receipt of Care Coordination Among High‐Risk Veterans in the Veterans Health Administration,” Medical Care 62, no. 8 (2024): 549–558, 10.1097/MLR.0000000000002020. 38967995 PMC11219070

[29] K. M. McDonald , S. J. Singer , S. S. Gorin , et al., “Incorporating Theory Into Practice: Reconceptualizing Exemplary Care Coordination Initiatives From the US Veterans Health Delivery System,” Journal of General Internal Medicine 34, no. Suppl 1 (2019): 24–29, 10.1007/s11606-019-04969-w. 31098965 PMC6542860

[30] C. L. Greenstone , J. Peppiatt , K. Cunningham , et al., “Standardizing Care Coordination Within the Department of Veterans Affairs,” Journal of General Internal Medicine 34, no. S1 (2019): 4–6, 10.1007/s11606-019-04997-6. 31098969 PMC6542928

[31] U.S. Department of Veterans Affairs , “Acute Hospitalization, Medicare,” 2023.

[32] U.S. Department of Veterans Affairs , “Acute Hospitalization, VA MedSAS,” 2023.

[33] U.S. Department of Veterans Affairs , “Acute Hospitalization, VA PIT,” 2023.

[34] Agency for Healthcare Research and Quality , “Prevention Quality Indicators Technical Specifications,” 2022.

[35] Agency for Healthcare Research and Quality , “Guide to Prevention Quality Indicators: Hospital Admission for Ambulatory Care Sensitive Conditions,” 2001.

[36] B. E. Flanagan , E. W. Gregory , E. J. Hallisey , J. L. Heitgerd , and B. Lewis , “A Social Vulnerability Index for Disaster Management,” J Homel Secur Emerg Manag 8, no. 1 (2011): 0000102202154773551792, 10.2202/1547-7355.1792.

[37] Centers for Disease Control and Prevention/Agency for Toxic Substances and Disease Registry , “Social Vulnerability Index (SVI),” 2023.

[38] P. L. Hebert , E. S. Wong , A. Reddy , et al., “Events Associated With Changes in Reliance on the Veterans Health Administration Among Medicare‐Eligible Veterans,” Medical Care 58, no. 8 (2020): 710–716, 10.1097/MLR.0000000000001328. 32265354

[39] P. C. Austin , “Using the Standardized Difference to Compare the Prevalence of a Binary Variable Between Two Groups in Observational Research,” Communications in Statistics: Simulation and Computation 38, no. 6 (2009): 1228–1234, 10.1080/03610910902859574.

[40] P. C. Austin , “Balance Diagnostics for Comparing the Distribution of Baseline Covariates Between Treatment Groups in Propensity‐Score Matched Samples,” Statistics in Medicine 28, no. 25 (2009): 3083–3107, 10.1002/sim.3697. 19757444 PMC3472075

[41] D. Yang and J. Dalton , “A Unified Approach to Measuring the Effect Size Between Two Groups Using SAS,” 2012.

[42] J. P. Fine and R. J. Gray , “A Proportional Hazards Model for the Subdistribution of a Competing Risk,” Journal of the American Statistical Association 94, no. 446 (1999): 496–509, 10.1080/01621459.1999.10474144.

[43] V. A. Smith , C. J. Coffman , and M. G. Hudgens , “Interpreting the Results of Intention‐To‐Treat, per‐Protocol, and as‐Treated Analyses of Clinical Trials,” Journal of the American Medical Association 326, no. 5 (2021): 433–434, 10.1001/jama.2021.2825. 34342631 PMC8985703

[44] N. Grafféo , A. Latouche , C. Le Tourneau , and S. Chevret , “Ipcwswitch: An R Package for Inverse Probability of Censoring Weighting With an Application to Switches in Clinical Trials,” Computers in Biology and Medicine 111 (2019): 103339, 10.1016/j.compbiomed.2019.103339. 31442762

[45] P. Cotterill , J. Bobula , and R. Connerton , “Comparison of Alternative Relative Weights for Diagnosis‐Related Groups,” Health Care Financing Review 7, no. 3 (1986): 37–51. 10311495 PMC4191521

[46] S. J. Bahr , S. Solverson , A. Schlidt , D. Hack , J. L. Smith , and P. Ryan , “Integrated Literature Review of Postdischarge Telephone Calls,” Western Journal of Nursing Research 36, no. 1 (2014): 84–104, 10.1177/0193945913491016. 23833254

[47] C. E. Woods , R. Jones , E. O'Shea , E. Grist , J. Wiggers , and K. Usher , “Nurse‐Led Postdischarge Telephone Follow‐Up Calls: A Mixed Study Systematic Review,” Journal of Clinical Nursing 28, no. 19–20 (2019): 3386–3399, 10.1111/jocn.14951. 31162748

[48] J. F. Fitzgerald , D. M. Smith , D. K. Martin , J. A. Freedman , and B. P. Katz , “A Case Manager Intervention to Reduce Readmissions,” Archives of Internal Medicine 154, no. 15 (1994): 1721–1729. 8042889

[49] H. Sjoberg , W. Liu , C. Rohs , et al., “Optimizing Care Coordination to Address Social Determinants of Health Needs for Dual‐Use Veterans,” BMC Health Services Research 22 (2022): 59, 10.1186/s12913-021-07408-x. 35022053 PMC8754195

[50] S. Spotswood , “Care Integration: Is VA Trying to Reinvent Something That Already Existed?” 2023.

[51] U.S. Department of Veterans Affairs , “Care Coordination and Integrated Case Management (CCICM) Implementation Guide,” 2024.

[52] U.S. Department of Veterans Affairs , “OVAHCS Unveils Groundbreaking Healthcare Initiative: Care Coordination and Integrated Case Management (CCICM),” 2024.

[53] D. Kansagara , J. Chiovaro , D. Kagen , et al., “Transitions of Care From Hospital to Home: An Overview of Systematic Reviews and Recommendations for Improving Transitional Care in the Veterans Health Administration,” 2015. 26312362

[54] R. Capp , G. J. Misky , R. C. Lindrooth , et al., “Coordination Program Reduced Acute Care Use and Increased Primary Care Visits Among Frequent Emergency Care Users,” Health Affairs 36, no. 10 (2017): 1705–1711, 10.1377/hlthaff.2017.0612. 28971914 PMC13075440

[55] M. Heo , K. Taaffe , A. Ghadshi , et al., “Effectiveness of Transitional Care Program Among High‐Risk Discharged Patients: A Quasi‐Experimental Study on Saving Costs, Post‐Discharge Readmissions and Emergency Department Visits,” International Journal of Environmental Research and Public Health 20, no. 23 (2023): 7136, 10.3390/ijerph20237136. 38063566 PMC10706296

[56] S. K. Patel , A. Miller , S. Chen , A. Lindsay , M. Gray , and Y.‐P. Su , “Transitional Care Management Visits to Improve Coordination of Care,” 2021. 10.37765/ajmc.2021.88622 33877780

[57] J. Pugh , L. S. Penney , P. H. Noël , et al., “Evidence Based Processes to Prevent Readmissions: More Is Better, a Ten‐Site Observational Study,” BMC Health Services Research 21 (2021): 189, 10.1186/s12913-021-06193-x. 33648491 PMC7919066

