# Supplementary material for: Risk of Hospital Readmissions and Association With Receipt of Post‐Hospitalization Care Coordination Services Among High‐Risk Veterans
Source: Health Serv Res. 2025 Sep 26;61(1):e70044. doi: 10.1111/1475-6773.70044 (PMC12857460; doi:10.1111/1475-6773.70044)
Supplement: Supplementary file 1 — Data S1: hesr70044‐sup‐0001‐TableS1.docx. [file HESR-61-0-s001.docx]

**SUPPLEMENTAL MATERIAL**

**TABLE OF CONTENTS**

Supplemental Table 1. Participant Characteristics By Post-Hospitalization Care Coordination Status, Before and After Matching

Supplemental Table 2. Comparisons of 30-Day Hospital Readmissions and Death by Post-Hospitalization Care Coordination Status, with Inversed Probability of Censoring Weights for Censoring Due to Cross-over to Treatment During Follow-up

Supplemental Table 3. Comparisons of 30-Day Hospital Readmissions and Death by Post-Hospitalization Care Coordination Status, with Medicare Advantage Exclusions

Supplemental Table 4. Comparisons of 30-Day Hospital Readmissions and Death by Post-Hospitalization Care Coordination Status, with Enhanced Matching Variables Related to Characteristics of Initial Hospitalization

Supplemental Table 5. Comparisons of 30-Day Hospital Readmissions and Death by Post-Hospitalization Care Coordination Status, Limited to Subgroup of Matched Treated and Comparators with COVID-19 as Initial Admitting Diagnosis

**Supplemental Table 1. Participant Characteristics By Post-Hospitalization Care Coordination Status, Before and After Matching**

|  | **Before Matching** | | | | | | | **After Matching** | | | | | | |
| --- | --- | --- | --- | --- | --- | --- | --- | --- | --- | --- | --- | --- | --- | --- |
|  |  |  | **Post-Hospitalization Care Coordination Status** | | | |  |  |  | **Post-Hospitalization Care Coordination Status** | | | |  |
| **Characteristic** | **Overall (N) N = 326,988** | **Overall (% or SD) 100%** | **No (N) N = 292,995** | **No (% or SD) 90%** | **Yes (N) N = 33,993** | **Yes (% or SD) 10%** | **SMD** | **Overall (N) N = 131,248** | **Overall  (% or SD) 100%** | **No (N) N = 99,634** | **No (% or SD) 76%** | **Yes (N) N = 31,614** | **Yes (% or SD) 24%** | **SMD** |
| Sex, No. (%) |  |  |  |  |  |  | 0.00 |  |  |  |  |  |  | 0.05 |
| Female | 19,017 | 5.8 | 17,045 | 5.8 | 1,972 | 5.8 |  | 4,519 | 3.4 | 3,198 | 3.2 | 1,321 | 4.2 |  |
| M | 307,971 | 94.2 | 275,950 | 94.2 | 32,021 | 94.2 |  | 126,729 | 96.6 | 96,436 | 96.8 | 30,293 | 95.8 |  |
| Age Group (years), No. (%) |  |  |  |  |  |  | 0.13 |  |  |  |  |  |  | 0.06 |
| <45 | 11,862 | 3.6 | 10,410 | 3.6 | 1,452 | 4.3 |  | 3,757 | 2.9 | 2,698 | 2.7 | 1,059 | 3.4 |  |
| 45-64 | 71,352 | 21.8 | 62,636 | 21.4 | 8,716 | 25.6 |  | 33,681 | 25.7 | 25,587 | 25.7 | 8,094 | 25.6 |  |
| 65-74 | 123,868 | 37.9 | 110,986 | 37.9 | 12,882 | 37.9 |  | 52,477 | 40.0 | 40,148 | 40.3 | 12,329 | 39.0 |  |
| 75-84 | 73,005 | 22.3 | 66,056 | 22.6 | 6,949 | 20.4 |  | 27,449 | 20.9 | 20,936 | 21.0 | 6,513 | 20.6 |  |
| 85+ | 46,901 | 14.3 | 42,907 | 14.6 | 3,994 | 11.8 |  | 13,884 | 10.6 | 10,265 | 10.3 | 3,619 | 11.5 |  |
| Mean CAN Score Category 1-year Pre-Hospitalization, No. (%) |  |  |  |  |  |  |  |  |  |  |  |  |  | 0.06 |
| (3, 81.6] | 83,496 | 25.5 | 76,835 | 26.2 | 6,661 | 19.6 |  | 27,498 | 21.0 | 21,266 | 21.3 | 6,232 | 19.7 |  |
| (81.6, 88.3] | 82,250 | 25.2 | 75,321 | 25.7 | 6,929 | 20.4 |  | 27,550 | 21.0 | 21,131 | 21.2 | 6,419 | 20.3 |  |
| (88.3, 94.5] | 81,457 | 24.9 | 72,935 | 24.9 | 8,522 | 25.1 |  | 33,061 | 25.2 | 25,106 | 25.2 | 7,955 | 25.2 |  |
| (94.5, 99] | 79,785 | 24.4 | 67,904 | 23.2 | 11,881 | 35.0 |  | 43,139 | 32.9 | 32,131 | 32.3 | 11,008 | 34.8 |  |
| Veteran’s Assigned VA Facility is a CCICM Early Adopter Site, No. (%) | 76,660 | 23.4 | 69,585 | 23.8 | 7,075 | 20.8 | 0.07 | 26,040 | 19.8 | 19,714 | 19.8 | 6,326 | 20.0 | 0.01 |
| Receipt of Care Coordination 1-year Pre-Hospitalization, No. (%) | 110,637 | 33.8 | 92,861 | 31.7 | 17,776 | 52.3 | 0.43 | 59,788 | 45.6 | 43,829 | 44.0 | 15,959 | 50.5 | 0.13 |
| Discharge Quarter, No. (%) |  |  |  |  |  |  | 0.03 |  |  |  |  |  |  | 0.01 |
| Q1 FY21 | 109,904 | 33.6 | 98,635 | 33.7 | 11,269 | 33.2 |  | 44,275 | 33.7 | 33,658 | 33.8 | 10,617 | 33.6 |  |
| Q2 FY21 | 83,178 | 25.4 | 74,582 | 25.5 | 8,596 | 25.3 |  | 33,027 | 25.2 | 25,056 | 25.2 | 7,971 | 25.2 |  |
| Q3 FY21 | 73,550 | 22.5 | 65,520 | 22.4 | 8,030 | 23.6 |  | 30,475 | 23.2 | 23,037 | 23.1 | 7,438 | 23.5 |  |
| Q4 FY21 | 60,356 | 18.5 | 54,258 | 18.5 | 6,098 | 17.9 |  | 23,471 | 17.9 | 17,883 | 18.0 | 5,588 | 17.7 |  |
| Setting of Initial Hospitalization, No. (%) |  |  |  |  |  |  | 1.12 |  |  |  |  |  |  | 0.11 |
| Medicare FFS | 57,106 | 17.5 | 56,560 | 19.3 | 546 | 1.6 |  | 2,665 | 2.0 | 2,159 | 2.2 | 506 | 1.6 |  |
| VA-Purchased | 100,859 | 30.8 | 98,650 | 33.7 | 2,209 | 6.5 |  | 11,517 | 8.8 | 9,406 | 9.4 | 2,111 | 6.7 |  |
| VA-Delivered | 169,023 | 51.7 | 137,785 | 47.0 | 31,238 | 91.9 |  | 117,066 | 89.2 | 88,069 | 88.4 | 28,997 | 91.7 |  |
| Race, No. (%) |  |  |  |  |  |  | 0.17 |  |  |  |  |  |  | 0.04 |
| American Indian/Alaska Native | 2,661 | 0.8 | 2,410 | 0.8 | 251 | 0.7 |  | 991 | 0.8 | 752 | 0.8 | 239 | 0.8 |  |
| Asian | 1,445 | 0.4 | 1,336 | 0.5 | 109 | 0.3 |  | 468 | 0.4 | 372 | 0.4 | 96 | 0.3 |  |
| Black/African American | 68,721 | 21.0 | 59,443 | 20.3 | 9,278 | 27.3 |  | 34,445 | 26.2 | 25,812 | 25.9 | 8,633 | 27.3 |  |
| Multiracial | 2,782 | 0.9 | 2,474 | 0.8 | 308 | 0.9 |  | 1,137 | 0.9 | 850 | 0.9 | 287 | 0.9 |  |
| Native Hawaiian/Pacific Islander | 2,416 | 0.7 | 2,224 | 0.8 | 192 | 0.6 |  | 859 | 0.7 | 678 | 0.7 | 181 | 0.6 |  |
| White | 245,339 | 75.0 | 221,885 | 75.7 | 23,454 | 69.0 |  | 91,791 | 69.9 | 69,968 | 70.2 | 21,823 | 69.0 |  |
| Other/Unknown Race Identity | 3,624 | 1.1 | 3,223 | 1.1 | 401 | 1.2 |  | 1,557 | 1.2 | 1,202 | 1.2 | 355 | 1.1 |  |
| Hispanic/Latino/a/-x Ethnicity, No. (%) |  |  |  |  |  |  | 0.01 |  |  |  |  |  |  | 0.02 |
| Non-Hispanic/Latino/a/-x | 312,029 | 95.4 | 279,627 | 95.4 | 32,402 | 95.3 |  | 124,617 | 95.0 | 94,488 | 94.8 | 30,129 | 95.3 |  |
| Hispanic/Latino/a/-x | 14,959 | 4.6 | 13,368 | 4.6 | 1,591 | 4.7 |  | 6,631 | 5.1 | 5,146 | 5.2 | 1,485 | 4.7 |  |
| Insurance/Coverage Status, No. (%) |  |  |  |  |  |  | 0.14 |  |  |  |  |  |  | 0.05 |
| VA Only | 35,826 | 11.0 | 31,412 | 10.7 | 4,414 | 13.0 |  | 16,328 | 12.4 | 12,373 | 12.4 | 3,955 | 12.5 |  |
| VA and Medicare | 253,222 | 77.4 | 228,662 | 78.0 | 24,560 | 72.3 |  | 97,818 | 74.5 | 74,695 | 75.0 | 23,123 | 73.1 |  |
| VA and Multiple | 37,940 | 11.6 | 32,921 | 11.2 | 5,019 | 14.8 |  | 17,102 | 13.0 | 12,566 | 12.6 | 4,536 | 14.4 |  |
| VA Priority Group, No. (%) |  |  |  |  |  |  | 0.14 |  |  |  |  |  |  | 0.05 |
| Group 1 | 142,293 | 43.5 | 128,535 | 43.9 | 13,758 | 40.5 |  | 54,649 | 41.6 | 41,821 | 42.0 | 12,828 | 40.6 |  |
| Group 2 | 16,531 | 5.1 | 14,775 | 5.0 | 1,756 | 5.2 |  | 6,817 | 5.2 | 5,181 | 5.2 | 1,636 | 5.2 |  |
| Group 3 | 30,185 | 9.2 | 27,133 | 9.3 | 3,052 | 9.0 |  | 12,322 | 9.4 | 9,441 | 9.5 | 2,881 | 9.1 |  |
| Group 4 | 11,813 | 3.6 | 10,416 | 3.6 | 1,397 | 4.1 |  | 4,676 | 3.6 | 3,414 | 3.4 | 1,262 | 4.0 |  |
| Group 5 | 86,570 | 26.5 | 75,992 | 25.9 | 10,578 | 31.1 |  | 38,772 | 29.5 | 29,020 | 29.1 | 9,752 | 30.9 |  |
| Group 6 | 3,785 | 1.2 | 3,407 | 1.2 | 378 | 1.1 |  | 1,579 | 1.2 | 1,215 | 1.2 | 364 | 1.2 |  |
| Group 7 | 8,323 | 2.6 | 7,420 | 2.5 | 903 | 2.7 |  | 3,510 | 2.7 | 2,652 | 2.7 | 858 | 2.7 |  |
| Group 8 | 27,488 | 8.4 | 25,317 | 8.6 | 2,171 | 6.4 |  | 8,923 | 6.8 | 6,890 | 6.9 | 2,033 | 6.4 |  |
| Psychoses, No. (%) | 63,821 | 19.5 | 55,563 | 19.0 | 8,258 | 24.3 | -0.13 | 27,645 | 21.1 | 20,333 | 20.4 | 7,312 | 23.1 | 0.07 |
| Depression, No. (%) | 145,058 | 44.4 | 129,056 | 44.1 | 16,002 | 47.1 | -0.06 | 58,821 | 44.8 | 44,151 | 44.3 | 14,670 | 46.4 | 0.04 |
| Alcohol Use Disorder, No. (%) | 71,562 | 21.9 | 62,349 | 21.3 | 9,213 | 27.1 | -0.14 | 32,497 | 24.8 | 24,150 | 24.2 | 8,347 | 26.4 | 0.05 |
| Substance Use Disorder, No. (%) | 49,741 | 15.2 | 42,876 | 14.6 | 6,865 | 20.2 | -0.15 | 23,126 | 17.6 | 17,024 | 17.1 | 6,102 | 19.3 | 0.06 |
| Elixhauser Score, Readmission, mean (SD) | 44.4 | 26.3 | 44.4 | 26.3 | 44.4 | 26.4 | 0.00 | 43.6 | 26.2 | 43.4 | 26.1 | 44.4 | 26.5 | 0.04 |
| COVID-19 Infection 1-year Pre-Hospitalization, No. (%) | 23,613 | 7.2 | 21189.0 | 7.2 | 2,424 | 7.1 | 0.00 | 9,395 | 7.2 | 7,144 | 7.2 | 2,251 | 7.1 | 0.00 |
| COVID-19 Vaccination Status, No. (%) |  |  |  |  |  |  | 0.01 |  |  |  |  |  |  | 0.02 |
| Fully Vaccinated | 104,234 | 31.9 | 93465.0 | 31.9 | 10,769 | 31.7 |  | 42,602 | 32.5 | 32,597 | 32.7 | 10,005 | 31.7 |  |
| Not Fully Vaccinated | 222,754 | 68.1 | 199530.0 | 68.1 | 23,224 | 68.3 |  | 88,646 | 67.5 | 67,037 | 67.3 | 21,609 | 68.4 |  |
| VA Hospitalization Count 1-year Pre-Hospitalization, mean (SD) | 0.3 | 0.9 | 0.3 | 0.8 | 0.5 | 1.1 | -0.20 | 0.5 | 1.0 | 0.5 | 1.0 | 0.5 | 1.1 | 0.03 |
| Medicare FFS Hospitalization Count 1-year Pre-Hospitalization, mean (SD) | 0.1 | 0.5 | 0.1 | 0.5 | 0.1 | 0.3 | 0.16 | 0.1 | 0.3 | 0.1 | 0.3 | 0.1 | 0.3 | 0.00 |
| VA-Purchased Hospitalization Count 1-year Pre-Hospitalization, mean (SD) | 0.2 | 0.6 | 0.2 | 0.6 | 0.1 | 0.5 | 0.09 | 0.1 | 0.5 | 0.1 | 0.5 | 0.1 | 0.5 | 0.01 |
| All Setting Hospitalization Count 1-year Pre-Hospitalization, mean (SD) | 0.6 | 1.2 | 0.6 | 1.2 | 0.7 | 1.3 | -0.06 | 0.6 | 1.3 | 0.6 | 1.3 | 0.7 | 1.3 | 0.02 |
| VA Primary Care Visit Count 1-year Pre-Hospitalization, mean (SD) | 10.6 | 8.9 | 10.5 | 8.8 | 11.3 | 9.6 | -0.08 | 11.3 | 9.2 | 11.3 | 9.0 | 11.3 | 9.7 | 0.00 |
| VA Mental Health Care Visit Count 1-year Pre-Hospitalization, mean (SD) | 6.6 | 18.0 | 5.9 | 16.5 | 12.4 | 27.2 | -0.29 | 8.3 | 19.9 | 7.5 | 18.3 | 10.8 | 23.9 | 0.15 |
| VA Specialty Care Visit Count 1-year Pre-Hospitalization, mean (SD) | 21.0 | 16.4 | 20.4 | 16.1 | 25.8 | 18.1 | -0.32 | 25.8 | 17.3 | 25.7 | 17.0 | 26.0 | 18.2 | 0.01 |
| VA Reliance, mean (SD) | 0.8 | 0.3 | 0.8 | 0.3 | 0.9 | 0.2 | -0.45 | 0.9 | 0.2 | 0.9 | 0.2 | 0.9 | 0.2 | 0.01 |
| Urban or Rural Residence, No. (%) |  |  |  |  |  |  | 0.14 |  |  |  |  |  |  | 0.03 |
| Urban | 221,241 | 67.7 | 196,290 | 67.0 | 24,951 | 73.4 |  | 94,859 | 72.3 | 71,699 | 72.0 | 23,160 | 73.3 |  |
| Rural/Highly Rural | 105,747 | 32.3 | 96,705 | 33.0 | 9,042 | 26.6 |  | 36,389 | 27.7 | 27,935 | 28.0 | 8,454 | 26.7 |  |
| Residence in PC-HPSA, No. (%) | 84,387 | 25.8 | 76,352 | 26.1 | 8,035 | 23.6 | 0.06 | 34,378 | 26.2 | 26,737 | 26.8 | 7,641 | 24.2 | 0.06 |
| Residence in MH-HPSA, No. (%) | 116,455 | 35.6 | 106,945 | 36.5 | 9,510 | 28.0 | 0.18 | 41,961 | 32.0 | 32,955 | 33.1 | 9,006 | 28.5 | 0.10 |
| Drive Time to Nearest VA (minutes), No. (%) |  |  |  |  |  |  | 0.13 |  |  |  |  |  |  | 0.04 |
| 0-10 | 93,835 | 28.7 | 82,859 | 28.3 | 10,976 | 32.3 |  | 40,337 | 30.7 | 30,290 | 30.4 | 10,047 | 31.8 |  |
| 11-20 | 119,609 | 36.6 | 106,682 | 36.4 | 12,927 | 38.0 |  | 49,822 | 38.0 | 37,773 | 37.9 | 12,049 | 38.1 |  |
| 21-30 | 56,920 | 17.4 | 51,561 | 17.6 | 5,359 | 15.8 |  | 21,589 | 16.5 | 16,542 | 16.6 | 5,047 | 16.0 |  |
| >30 | 56,624 | 17.3 | 51,893 | 17.7 | 4,731 | 13.9 |  | 19,500 | 14.9 | 15,029 | 15.1 | 4,471 | 14.1 |  |
| Drive Distance to VA (miles), No. (%) |  |  |  |  |  |  | 0.15 |  |  |  |  |  |  | 0.05 |
| 0-5 | 88,868 | 27.2 | 78,207 | 26.7 | 10,661 | 31.4 |  | 38,715 | 29.5 | 28,960 | 29.1 | 9,755 | 30.9 |  |
| 6-10 | 78,545 | 24.0 | 69,884 | 23.9 | 8,661 | 25.5 |  | 33,205 | 25.3 | 25,144 | 25.2 | 8,061 | 25.5 |  |
| 11-20 | 83,047 | 25.4 | 74,721 | 25.5 | 8,326 | 24.5 |  | 32,833 | 25.0 | 25,025 | 25.1 | 7,808 | 24.7 |  |
| 21-40 | 58,317 | 17.8 | 53,381 | 18.2 | 4,936 | 14.5 |  | 20,561 | 15.7 | 15,899 | 16.0 | 4,662 | 14.8 |  |
| >40 | 18,211 | 5.6 | 16,802 | 5.7 | 1,409 | 4.1 |  | 5,934 | 4.5 | 4,606 | 4.6 | 1,328 | 4.2 |  |
| SVI, Socioeconomic Status, mean (SD) | 0.6 | 0.3 | 0.6 | 0.3 | 0.6 | 0.3 | -0.02 | 0.6 | 0.3 | 0.6 | 0.3 | 0.6 | 0.3 | 0.06 |
| SVI, Household Characteristics, mean (SD) | 0.5 | 0.3 | 0.5 | 0.3 | 0.5 | 0.3 | 0.03 | 0.5 | 0.3 | 0.5 | 0.3 | 0.5 | 0.3 | 0.04 |
| SVI, Racial and Ethnic Minority Status, mean (SD) | 0.7 | 0.3 | 0.7 | 0.3 | 0.7 | 0.3 | -0.11 | 0.7 | 0.3 | 0.7 | 0.2 | 0.7 | 0.3 | 0.03 |
| SVI, Housing Type and Transportation, mean (SD) | 0.6 | 0.2 | 0.6 | 0.2 | 0.6 | 0.2 | -0.06 | 0.6 | 0.2 | 0.6 | 0.2 | 0.6 | 0.2 | 0.01 |
| County Long-Term Care Bed Count, mean (SD) | 3,596.3 | 6414.9 | 3473.1 | 6253.3 | 4658.0 | 7585.0 | -0.17 | 4740.6 | 7,913.7 | 4756.7 | 7999.3 | 4689.8 | 7637.5 | 0.01 |
| County Hospital Bed Count, mean (SD) | 2,180.1 | 3635.4 | 2110.4 | 3539.9 | 2780.4 | 4326.5 | -0.17 | 2841.5 | 4,367.8 | 2853.1 | 4370.9 | 2805.2 | 4357.9 | 0.01 |
| Length of Initial Hospitalization (days), No. (%) |  |  |  |  |  |  | 0.16 |  |  |  |  |  |  | 0.14 |
| 1 | 68,121 | 20.8 | 60,677 | 20.7 | 7,444 | 21.9 |  | 31,907 | 24.3 | 24,844 | 24.9 | 7,063 | 22.3 |  |
| 2 | 63,745 | 19.5 | 58,140 | 19.8 | 5,605 | 16.5 |  | 24,190 | 18.4 | 18,839 | 18.9 | 5,351 | 16.9 |  |
| 3-7 | 140,478 | 43.0 | 126,828 | 43.3 | 13,650 | 40.2 |  | 53,622 | 40.9 | 40,790 | 40.9 | 12,832 | 40.6 |  |
| 8-14 | 36,729 | 11.2 | 32,008 | 10.9 | 4,721 | 13.9 |  | 14,902 | 11.4 | 10,671 | 10.7 | 4,231 | 13.4 |  |
| 15+ | 17,915 | 5.5 | 15,342 | 5.2 | 2,573 | 7.6 |  | 6,627 | 5.1 | 4,490 | 4.5 | 2,137 | 6.8 |  |
| Primary Diagnosis of COVID-19 for Initial Hospitalization, No. (%) | 27,005 | 8.3 | 24,435 | 8.3 | 2,570 | 7.6 | 0.03 | 9,988 | 7.6 | 7,596 | 7.6 | 2,392 | 7.6 | 0.00 |

Supplementary Table 1 Footnote: Veterans Integrated Service Network and state of residence omitted for table brevity. Abbreviations: SMD: absolute Standard Mean Difference; VA: Veterans Health Administration; CCICM: Care Coordination and Integrated Case Management; CAN: Care Assessment of Need; CPT: Current Procedural Terminology; PC-HPSA: primary care health professional shortage area; MH-HPSA: mental health health professional shortage area; SVI: Social Vulnerability Index.

**Supplemental Table 2. Comparisons of 30-Day Hospital Readmissions and Death by Post-Hospitalization Care Coordination Status, with Inversed Probability of Censoring Weights for Censoring Due to Cross-over to Treatment During Follow-up**

|  | **Cumulative Incidence** | | | | |  |  |  |
| --- | --- | --- | --- | --- | --- | --- | --- | --- |
|  | **Overall** | **Post-Hospitalization Care Coordination Status** | | **Unadjusted Percentage Point Difference** | | **Adjusted Subhazard Ratio (aSHR)** | | |
|  | **N=131,248** | **Yes**  **N=31,614 (24%)** | **No**  **N=99,634 (76%)** | **Estimate** | **95% CI** | **Estimate** | **95% CI** |  |
| *All-Cause Readmission | 13.8% | 15.9% | 13.1% | 2.77 | 2.31, 3.22 | 1.09 | 1.05, 1.13 |  |
| **ACSC Readmission | 2.1% | 2.3% | 2.0% | 0.22 | 0.04, 0.41 | 1.00 | 0.92, 1.09 |  |
| ***Death | 2.3% | 3.7% | 1.84% | 1.86 | 1.64, 2.08 | 1.96 | 1.82, 2.12 |  |

Supplemental Table 2 Footnote: Unstabilized censoring weights were calculated as the probability of being censored based on a Cox proportional hazards regression model that included treatment and 30 demographic, geographic, healthcare utilization, and clinical factors. We examined weights for extreme values (range: 1-16.166, mean: 1.108, standard deviation: 0.137, median: 1.071 , and interquartile range: 1.050-1.141). All models were adjusted by matching variables with SMDs >0.1. *All-cause readmission aSHR and 95% CI estimated in a model with death as a competing risk; **ACSC readmission aSHR and 95% CI estimated in a model with non-ACSC readmission and death as competing risks. *** Death aSHR and 95% CI are the same in both models. Abbreviations: ACSC: Ambulatory Care-Sensitive Condition; aSHR: Adjusted Sub-Hazard Ratio; CI: Confidence Interval.

**Supplemental Table 3. Comparisons of 30-Day Hospital Readmissions and Death by Post-Hospitalization Care Coordination Status, with Medicare Advantage Exclusions**

|  | **Cumulative Incidence** | | | | |  | |
| --- | --- | --- | --- | --- | --- | --- | --- |
|  | **Overall** | **Post-Hospitalization Care Coordination Status** | | **Unadjusted Percentage Point Difference** | | **Adjusted Subhazard Ratio (aSHR)** | |
|  | **N=86,964** | **Yes**  **N=23,612 (27%)** | **No**  **N=63,352 (73%)** | **Estimate** | **95% CI** | **Estimate** | **95% CI** |
| *All-Cause Readmission | 14.3% | 16.2% | 13.6% | 2.54 | 2.00, 3.08 | 1.05 | 1.01, 1.09 |
| **ACSC Readmission | 2.1% | 2.2% | 2.1% | 0.16 | -0.07, 0.38 | 0.98 | 0.88, 1.08 |
| ***Death | 2.3% | 3.6% | 1.8% | 1.84 | 1.58, 2.11 | 2.03 | 1.85, 2.23 |

Supplemental Table 3 Footnote: Treated and their matched comparators with Medicare Advantage were excluded, as were individual comparators whose matched treated did not have Medicare Advantage. If exclusion of matched comparators with Medicare Advantage resulted in a treated with no matches, that treated was also excluded. All models were adjusted by matching variables with SMDs >0.1. *All-cause readmission aSHR and 95% CI estimated in a model with death as a competing risk; **ACSC readmission aSHR and 95% CI estimated in a model with non-ACSC readmission and death as competing risks. *** Death aSHR and 95% CI are the same in both models. Abbreviations: ACSC: Ambulatory Care-Sensitive Condition; aSHR: Adjusted Sub-Hazard Ratio; CI: Confidence Interval.

**Supplemental Table 4. Comparisons of 30-Day Hospital Readmissions and Death by Post-Hospitalization Care Coordination Status, with Enhanced Matching Variables Related to Characteristics of Initial Hospitalization**

|  | **Cumulative Incidence** | | | | |  | |
| --- | --- | --- | --- | --- | --- | --- | --- |
|  | **Overall** | **Post-Hospitalization Care Coordination Status** | | **Unadjusted Percentage Point Difference** | | **Adjusted Subhazard Ratio (aSHR)** | |
|  | **N=89,408** | **Yes**  **N=25,309 (28%)** | **No**  **N=64,099 (72%)** | **Estimate** | **95% CI** | **Estimate** | **95% CI** |
| *All-Cause Readmission | 14.2% | 15.7% | 13.6% | 2.08 | 1.56, 2.61 | 1.01 | 0.98, 1.05 |
| **ACSC Readmission | 2.2% | 2.3% | 2.1% | 0.22 | 0.01, 0.44 | 0.99 | 0.90, 1.10 |
| ***Death | 2.2% | 3.4% | 1.7% | 1.72 | 1.48, 1.97 | 1.92 | 1.75, 2.11 |

Supplemental Table 4 Footnote: Matching variables for competing risk models included the 8 exact match and 32 propensity score match variables that were included in matching in primary analyses, as well as three additional exact match variables, including: 1) whether the initial hospitalization involved an intensive care unit (ICU) stay, 2) quartiles of Diagnostic Related Group (DRG) weights for the initial hospital stay, and 3) whether the initial hospital discharge occurred on a weekday or weekend. All models were adjusted by matching variables with SMDs >0.1. *All-cause readmission aSHR and 95% CI estimated in a model with death as a competing risk; **ACSC readmission aSHR and 95% CI estimated in a model with non-ACSC readmission and death as competing risks. *** Death aSHR and 95% CI are the same in both models. Abbreviations: ACSC: Ambulatory Care-Sensitive Condition; aSHR: Adjusted Sub-Hazard Ratio; CI: Confidence Interval.

**Supplemental Table 5. Comparisons of 30-Day Hospital Readmissions and Death by Post-Hospitalization Care Coordination Status, Limited to Subgroup of Matched Treated and Comparators with COVID-19 as Initial Admitting Diagnosis**

|  | **Cumulative Incidence** | | | | |  | |
| --- | --- | --- | --- | --- | --- | --- | --- |
|  | **Overall** | **Post-Hospitalization Care Coordination Status** | | **Unadjusted Percentage Point Difference** | | **Adjusted Subhazard Ratio (aSHR)** | |
|  | **N=2,850** | **Yes**  **N=866 (30%)** | **No**  **N=1,984 (70%)** | **Estimate** | **95% CI** | **Estimate** | **95% CI** |
| *All-Cause Readmission | 17.7% | 20.0% | 16.7% | 3.29 | 0.083, 6.50 | 1.16 | 0.96, 1.39 |
| **ACSC Readmission | 1.4% | 2.0% | 1.1% | 0.85 | -0.26, 1.97 | 1.51 | 0.79, 2.89 |
| ***Death | 3.2% | 4.5% | 2.6% | 1.88 | 0.25, 3.52 | 1.44 | 0.92, 2.27 |

Supplemental Table 5 Footnote: Analysis limited to treated and matched comparators with a primary diagnosis of COVID-19 for their initial hospitalization. All models were adjusted by matching variables with SMDs >0.1. *All-cause readmission aSHR and 95% CI estimated in a model with death as a competing risk; **ACSC readmission aSHR and 95% CI estimated in a model with non-ACSC readmission and death as competing risks. *** Death aSHR and 95% CI are the same in both models. Abbreviations: ACSC: Ambulatory Care-Sensitive Condition; aSHR: Adjusted Sub-Hazard Ratio; CI: Confidence Interval.
